# Rage gene -374T/A polymorphism in South Indian diabetic nephropathy

**DOI:** 10.6026/973206300212288

**Published:** 2025-08-31

**Authors:** Ramesh Babu Murikipudi, Lakshmiprabha S., Pravallika P., Ponnudhali D., Rangabashyam S.R., Rangarajan R.

**Affiliations:** 1 Department of Biochemistry, Vinayaka Mission's Kirupananda Variyar Medical College & Hospital, Salem, Vinayaka Misson's Research Foundation (DU), Salem, Tamilnadu, India; 2 Department of Biochemistry, KMMC Medical College and Hospital, Muttom, Kanyakumari., St. Joseph University, Bengaluru, India; 3 Department of Physiology, Shridevi institute of medical sciences and research hospital Tumkur, Karnataka, India; 4 Department of General medicine, Vinayaka Mission's Kirupananda Variyar Medical College & Hospital, Salem, India; 5 Department of Biochemistry, Government Mohan Kumaramangalam Medical College Hospital, Shevapet, Salem, India

**Keywords:** Rage gene, Blood parameters, glycemic index, gene polymorphism

## Abstract

Diabetic nephropathy (DN) is a major complication of type 2 diabetes mellitus (T2DM) and genetic variants like RAGE -374T/A may influence disease susceptibility. Therefore, it is of interest to analyze the association between RAGE -374T/A polymorphism and DN in T2DM patients from Kolar, Karnataka. Genotyping was done by PCR-RFLP and clinical parameters were compared. No significant association was found between the polymorphism and DN (p > 0.05), though DN patients showed elevated HbA1c, microalbuminuria and dyslipidemia. These findings suggest metabolic factors, rather than this specific RAGE variant, are more strongly linked to DN risk in this population.

## Background:

Type 2 diabetes mellitus (T2DM) is a chronic metabolic disorder marked by sustained hyperglycemia due to insulin resistance or impaired secretion, affecting over 400 million people globally [1]. The global burden of diabetes is rising, particularly in low- and middle-income countries, with India being among the top three nations in prevalence [2]. Diabetic nephropathy (DN), a common microvascular complication of T2DM, is a major cause of end-stage renal disease and increases cardiovascular risk [3]. DN pathogenesis is multifactorial, involving poor glycemic control, hypertension and genetic predisposition [4]. Advanced glycation end-products (AGEs), formed through non-enzymatic glycation of proteins or lipids during hyperglycemia, contribute to renal damage by binding to the receptor for AGEs (RAGE) expressed on renal and vascular tissues [5]. AGE-RAGE interaction activates oxidative stress and proinflammatory signaling pathways including NF-κB and TGF-β, promoting glomerular dysfunction and fibrosis [6]. The RAGE gene, located on chromosome 6p21.3, contains several functional single nucleotide polymorphisms (SNPs) in its promoter and coding regions that may influence its expression [7]. Among these, the -374T/A promoter polymorphism (rs1800624) is proposed to modulate RAGE transcriptional activity, potentially altering susceptibility to DN [8]. Although prior studies have evaluated RAGE polymorphisms such as Gly82Ser and -429T/C in Indian and other ethnic populations, data on -374T/A remain limited, especially in South Indian cohorts [7, 8]. Therefore, it is of interest to investigate the association between the RAGE -374T/A polymorphism and diabetic nephropathy in T2DM patients from the Kolar region of Karnataka, India.

## Materials and Methods:

This comparative cross-sectional study was conducted among T2DM patients residing in and around the Kolar region of Karnataka. Participants were selected based on expert referrals from relevant clinical departments. A total of 150 individuals were enrolled, including 100 patients diagnosed with T2DM for more than 5 years, as defined by the 2013 American Diabetes Association (ADA) criteria-fasting plasma glucose ≥126 mg/dL, postprandial glucose ≥200 mg/dL, or a confirmed clinical history of diabetes [9]. All participants were over the age of 40.

## Study Groups:

Participants were classified into three groups based on their urine albumin-to-creatinine ratio (UACR): Group A consisted of 50 age- and sex-matched healthy controls without diabetes. Group B included 50 T2DM patients without nephropathy (UACR <30 mg/g). Group C comprised of 50 T2DM patients with nephropathy (UACR ≥30 mg/g in at least 2 of 3 fasting urine samples collected over 2 months, or those on dialysis/kidney transplantation) [9]. The sample size was calculated using OpenEpi software, based on anticipated frequency and confidence levels. Ethical approval was obtained from the Institutional Ethics Committee and written informed consent was secured from all participants.

## Exclusion criteria:

Subjects with type 1 diabetes, diabetes duration <5 years, urinary tract infections, hematuria, non-diabetic renal disease, endocrine disorders, malignancies, obesity, pregnancy, or terminal illnesses were excluded from the study [10, 11].

## Sample collection and processing:

A detailed clinical history was obtained via a standardized questionnaire. Early morning fasting venous blood and sterile midstream urine samples were collected. To minimize the influence of medication, samples were drawn before administration of morning doses. A total of 8 mL of venous blood was collected as follows: 3 mL in EDTA tubes (for HbA1c and DNA extraction), 2 mL in fluoride vials (for fasting glucose), 3 mL in clot-activator tubes (for serum analysis). All non-EDTA blood and urine samples were centrifuged at 4000 rpm for 6 minutes at room temperature and the supernatants were stored at -80°C. Routine biochemical parameters-including fasting glucose, lipid profile and renal markers-were assessed in the Clinical Biochemistry Laboratory [12].

## Primer design:

Primers were designed using the NCBI reference genome (GRCh38.p14, annotation release RS_2023_03). The primers used for amplification of the RAGE -374T/A polymorphic region were: Forward: 5'-GGG GCA GTT CTC TCC TCA CT-3', Reverse: 5'-GGT TCA GGC CAG ACT GTT GT-3' [13].

## DNA extraction and genotyping:

Genomic DNA was extracted from EDTA blood samples using the Qiagen DNA extraction kit following the manufacturer's protocol. DNA was preserved in TAE buffer at -80°C. Purity was checked using a Nanodrop spectrophotometer (Nabi, MicroDigital), with an acceptable A260/A280 ratio between 1.8 and 2.0. Integrity was verified by electrophoresis on 1.2% agarose gel [13].

## PCR Amplification:

Each 50 µL PCR reaction consisted of 25 µL of 10X Master Mix (Taq polymerase, buffer, MgCl_2_, dNTPs), 5 µL genomic DNA, 1.25 µL each of forward and reverse primers, 17.5 µL nuclease-free water. Amplification was performed in a Veriti Thermal Cycler (Applied Biosystems, Thermo Fisher) with the following cycling conditions: an Initial denaturation at 95°C for 1 min followed by 35 cycles of 95°C for 15 s (denaturation), 55°C for 15 s (annealing), 72°C for 30 s (extension), Final extension at 72°C for 7 min [14].

## RFLP genotyping:

The 220 bp PCR product was digested overnight at 37°C using MfeI (Thermo Scientific) to detect the -374T/A polymorphism. The digested products were visualized on 2% agarose gel stained with ethidium bromide under UV light. Banding patterns were interpreted as: TT (wild type): 128 bp and 75 bp; AA (mutant): 203 bp; TA (heterozygous): 203 bp, 128 bp and 75 bp [15].

## Statistical analysis:

Statistical analysis was conducted using SPSS version 21.0 (IBM Corp., Armonk, NY) on a Windows 11 platform. Descriptive statistics were presented as mean ± standard deviation (SD) for continuous variables and as percentages (%) for categorical variables. One-way ANOVA followed by Tukey's post-hoc test was employed to compare continuous variables among the three study groups. Genotype and allele frequencies were analyzed using the Chi-square (χ^2^) test. Hardy-Weinberg equilibrium (HWE) was assessed to verify the distribution of genotypes. Odds ratios (OR) with 95% confidence intervals (CI) were calculated to estimate the strength of association between genotypes and diabetic nephropathy. A p-value <0.05 was considered statistically significant [16].

## Results:

The study enrolled 150 participants divided equally into three groups: Group A (Healthy Controls, n = 50), Group B (Type 2 Diabetes Mellitus, n = 50) and Group C (Diabetic Nephropathy, n = 50). The mean ages were comparable among the groups - Group A: 52.16 ± 7.29 years, Group B: 57.34 ± 7.87 years and Group C: 59.7 ± 8.34 years. The gender distribution showed a predominance of males in all groups (Group A: 60%, Group B: 74%, Group C: 70%). Fasting blood sugar (FBS), post-prandial blood sugar (PPBS) and HbA1c levels were significantly higher in both Group B and Group C compared to Group A (ANOVA; F values: FBS = 213, PPBS = 654, HbA1c = 233; p < 0.00001). Post hoc analysis confirmed significant differences between the groups (p < 0.001). Notably, HbA1c levels showed no significant difference between Group B and Group C (p = 0.177), suggesting similar glycemic control in these groups (Table 1 - see PDF). Data expressed as Mean ± SD. Comparisons were performed using One-way ANOVA followed by Post Hoc analysis. Significance considered at p < 0.05. N= Normal range; HC- Healthy controls, T2DM- Type 2 Diabetes mellitus, DN-Diabetic nephropathy P1- group A versus group B, P2- group A versus group C; P3- group B versus group C. N= Normal range; HC- Healthy controls, T2DM- Type 2 Diabetes mellitus, DN-Diabetic nephropathy P1- group A versus group B, P2- group A versus group C; P3- group B versus group C. Total cholesterol, triglycerides, LDL and VLDL levels were significantly elevated in Group C compared to Groups A and B (ANOVA; Total Cholesterol F = 165.2, Triglycerides F = 291.18, LDL F = 135.71, VLDL F = 291.18; all p < 0.00001). HDL levels were significantly lower in Group C (F = 18.06, p < 0.00001). These findings indicate worsening dyslipidemia with disease progression (Table 2 - see PDF). HC- Healthy controls, T2DM- Type 2 Diabetes mellitus, DN-Diabetic nephropathy, TT- Wild type, TA- heterozygous for mutant, AA-mutant band, P1- group A versus group B, P2- group A versus group C; P3- group B versus group C.

Table 3 (see PDF) & 4 (see PDF) Data expressed as n (%). Chi-square test applied. P < 0.05 considered significant. The distribution of -374T/A RAGE gene polymorphism genotypes showed no significant difference across the three groups (Chi-square test: χ^2^ = 1.915, p = 0.551). The TT genotype was most prevalent in all groups (Group A: 74%, Group B: 70%, Group C: 64%) and the homozygous AA genotype was absent in all groups. The allele frequencies also did not differ significantly (χ^2^ = 0.976, p = 0.614). Data expressed as Odds Ratio (95% CI). Association tested using Chi-square test with p < 0.05 considered significant. UCL- Upper confidence limit, LCL- Lower confidence limit, OR- Odds ratio, CI- confidence interval Odds ratio (OR) analysis revealed a significant association between the presence of the TA genotype and T2DM (Group B) compared to healthy controls (Group A) (OR = 1.2198; 95% CI: 0.5086-2.9254; p = 0.0289). The comparison between Groups A and C showed no statistical significance (OR = 1.6010; p = 0.082). However, a borderline significance was observed between Groups B and C (OR = 1.3125; p = 0.0556), suggesting a potential increasing trend of the TA genotype in DN patients (Table 5 - see PDF). Student t test was used to find the association between the genotype and HBA1C and Urine Microalbumin and the results was found to be significant with P<0.05. Patients carrying the TA genotype exhibited significantly higher HbA1c levels compared to those with TT genotype (HbA1c: 14 ± 1.9 vs. 9.5 ± 1.0; Student's t-test; t = 2.67, p = 0.0102). Urine microalbumin levels were higher in the TA group but did not reach statistical significance (Table 6 - see PDF).

## Discussion:

This study investigated the association between the RAGE -374T/A gene polymorphism and diabetic nephropathy (DN) in T2DM patients from the Kolar region of Karnataka. The genotypic distribution (TT, TA, AA) did not differ significantly between DN and non-DN groups, indicating no significant association between this SNP and DN risk. However, elevated HbA1c, urine microalbumin and dyslipidemia were strongly associated with DN, reinforcing their clinical utility in identifying high-risk patients. RAGE plays a key role in diabetic complications by interacting with advanced glycation end-products (AGEs), which trigger oxidative stress and inflammatory pathways that lead to endothelial and renal damage [7]. The -374T/A polymorphism lies within the RAGE gene promoter and is proposed to influence transcription factor binding and gene expression [16]. However, the absence of a significant association in our cohort aligns with studies in Brazilian and German populations that also found no correlation between this SNP and DN [17, 18]. A meta-analysis by Liu *et al.* concluded that the -374T/A SNP does not consistently associate with DN across ethnic groups, suggesting that population-specific genetic effects and environmental modifiers are critical [12]. Other RAGE variants-such as -429T/C and Gly82Ser-have shown stronger links to diabetic complications in multiple ethnicities, indicating that -374T/A may be less informative as a standalone marker [19]. In contrast, elevated HbA1c in DN patients reflects persistent hyperglycemia, which promotes AGE accumulation, glomerular basement membrane thickening and mesangial expansion-hallmarks of diabetic kidney injury [7]. The DCCT and UKPDS trials have firmly demonstrated that improved glycemic control reduces the risk of microvascular complications, including nephropathy [20]. Microalbuminuria, a marker of early glomerular damage, was significantly higher among DN patients in our study. Increased UACR is widely accepted as an early predictor of DN and cardiovascular risk and regular UACR screening remains essential in diabetic care [9].

Dyslipidemia, observed as higher triglycerides and LDL with lower HDL levels, was more prevalent in the DN group. This supports the role of lipid-induced nephrotoxicity in promoting glomerulosclerosis and tubulointerstitial injury [21]. However, the lack of association between dyslipidemia and the -374T/A genotype implies that lipid damage pathways may act independently of this RAGE variant. The study's limitation is its focus on a single SNP, which may not capture broader genetic influences. Gene-environment interactions are particularly relevant in heterogeneous populations such as South Indians, where lifestyle, ancestry and environmental exposures vary widely [22]. Future studies incorporating polygenic risk scores, haplotypes and environmental data will better define genetic susceptibility to DN [23]. In summary, while the -374T/A RAGE polymorphism was not associated with DN in this South Indian cohort, glycemic control, microalbuminuria and dyslipidemia were significantly linked to disease status. These findings emphasize the importance of routine biochemical markers and the need for expanded genetic research in diabetic nephropathy. In conclusion, the RAGE -374T/A polymorphism was not associated with DN in this cohort. However, biochemical parameters such as HbA1c, UACR and lipid levels were significantly correlated with DN status, reaffirming their clinical utility. These results emphasize the need for comprehensive genetic studies involving multiple SNPs, haplotypes and environmental modulators to better elucidate the complex etiology of diabetic nephropathy in Indian populations.
